# What makes reversal: examining the moderating effect of being a state functionary on occupational status and depression among middle-aged and older people in China

**DOI:** 10.1093/joccuh/uiaf008

**Published:** 2025-03-25

**Authors:** Haoran Li, Tao Xie, Jingya Zhang, Bin Zhu, Ning Zhang, Ying Mao

**Affiliations:** School of Public Policy and Administration, Xi'an Jiaotong University, Xi'an, Shaanxi, China; School of Public Policy and Administration, Xi'an Jiaotong University, Xi'an, Shaanxi, China; School of Public Policy and Administration, Xi'an Jiaotong University, Xi'an, Shaanxi, China; School of Public Health and Emergency Management, Southern University of Science and Technology, Shenzhen, Guangdong, China; School of Public Policy and Administration, Xi'an Jiaotong University, Xi'an, Shaanxi, China; School of Public Policy and Administration, Xi'an Jiaotong University, Xi'an, Shaanxi, China

**Keywords:** depression, occupational status, aging, moderating effect, state functionary

## Abstract

**Objectives:**

This study measured occupational status from the perspectives of occupational socioeconomic status, employment relationship, and class level, aiming to examine the effect of occupational status on depression among middle-aged and elderly people in China and determine whether being a state functionary plays a moderating role.

**Methods:**

Panel data from the China Family Panel Studies (*n* = 28 645) were used and the year fixed-effects model was adopted. The 2-way interaction terms “state functionary × occupational status (the International Socioeconomic Index of Occupational Status [ISEI], the Standard International Occupational Prestige Scale [SIOPS], and the Erikson and Goldthorpe class categories [EGP])” were added to examine whether being a state functionary could moderate the relationship between occupational status and depression.

**Results:**

Occupational status was negatively correlated with depression (ISEI: coefficient = −0.03; 95% CI, −0.04 to −0.02; SIOPS: coefficient = −0.01; 95% CI, −0.02 to −0.002; EGP: coefficient = 0.12; 95% CI, 0.08 to 0.15). The 2-way interaction terms “state functionary × occupational status (ISEI/SIOPS/EGP)” were significant among all middle-aged and older participants. The 2-way interaction terms were also significant in the educated and urban subgroups.

**Conclusions:**

Higher occupational status is a protective factor against depression among middle-aged and older Chinese adults. However, being a state functionary can reverse the relationship between occupational status and depression. We found that the higher the occupational status of state functionaries, the more severe their depression. We also found a moderating effect of being a state functionary in the educated and urban subgroups.

## Teaser

Key points

1) The association between high occupational status and less severe depression among middle-aged and older adults was significant.
2) Being a state functionary may result in a positive relationship between higher occupational status and greater depressive symptoms among middle-aged and older adults.
3) The moderating effect of state functionality was significant only in the educated and urban subgroups.

## 1. Introduction

Depression, the most harmful and common mental illness, has an increasing impact on healthy aging.^1^ According to the World Health Organization, the global incidence of depressive symptoms among older adults varies from 10% to 20% globally, and depression is more prevalent among older adults in developing countries than in developed ones.^2^ According to the Chinese Center for Disease Control and Prevention, more than 30% of older adults are at high risk of being diagnosed with depression, especially in rural areas.^3^ The high incidence of depression creates high costs for depression treatment, with direct costs reaching US $986 million and indirect costs reaching US $5278 million.^4^ China faces the most serious aging problems and it is necessary to thoroughly study depression among middle-aged and older adults to promote healthy aging.^5^

Depression among middle-aged and older people is related to physical function and demographic and social factors.^6^^,^^7^ As one of the most important demographic and social factors,^8^ occupational status significantly affects depression.^9^ However, the effect of occupational status on depression remains inconclusive, with the extant literature presenting 3 main views.^8^ First, some propose that occupational status and depression are negatively correlated; that is, people with high occupational status are less depressed.^9^^,^^10^ Studies have shown that the effect of various factors (eg, high job stress, long working hours, low pay, low autonomy, and poor mental health)^11^^,^^12^ on depression are more significant among people with low occupational status.^9^ In contrast, other studies have reported the opposite, suggesting that, in some cases, high occupational status predicts a higher risk of depression. For example, one study found that the living environment of people with high occupational status can lead to high levels of peer pressure, making people with high occupational status more prone to depression.^13^ High occupational status also tends to lead to conflicts between family and work, resulting in greater stress; however, this link may be influenced by gender and educational attainment.^14^ Third, the effect of occupational status on depression may be influenced by other factors. For example, the benefits or changes in lifestyle owing to occupation may influence the effect of occupational status on depression.^8^ For older people, generous pensions provided by jobs can buffer against the negative effects on depression of low occupational status.^15^ Moreover, the frequency of older people’s internet use influenced by previous work experience can inhibit the association between low occupational status and more severe depressive symptoms; however, this mechanism was only found in urban older people and not in rural older people.^8^ A specific job can also negatively impact an individual while simultaneously bringing benefits; for instance, high-paying jobs can cause high stress in work and family.^16^ Therefore, determining how specific occupations influence the effect of occupational status on depression is difficult.

Chinese state functionaries are a special group. They work in public, government, and social organizations to provide broad and common services to society.^17^ Due to the roles of state functionaries in the running of society, the public believes that such jobs are respectable. Moreover, their salaries are directly paid by state finance without the risk of unemployment, and the state provides them with various benefits such as housing subsidies, medical subsidies, and pensions.^18^ However, state functionaries face low pay, high work pressure, complex working environments, and other difficulties.^19^^,^^20^ Owing to these aspects of their jobs there is no consensus on whether being a state functionary alleviates or worsens depression. On the one hand, state functionaries may face greater mental health problems due to occupational stress caused by the special nature of their work.^21^ Their responsibilities may make them more likely to be depressed than members of the general population.^22^ However, on the other hand, research has suggested that a lower risk of unemployment, more stable income, and fewer working hours may prevent state functionaries from becoming depressed.^18^^,^^23^

Faced with the pressure of an aging population in China, we need to pay attention to depression among middle-aged and older adults. As an important factor that may affect depression, the influencing mechanism of occupational status is important for developing strategies to relieve depressive symptoms in these age groups. However, the effect of occupational status on depression has not been well established, and due to the coexistence of benefits and burdens, how serving as a state functionary affects the relationship between occupational status and depression in middle-aged and older adults remains unclear.

Using panel data from the China Family Panel Studies (CFPS), this study focused on the effect of occupational status on depression among middle-aged and older people and analyzed how serving as a state functionary affects this relationship. Based on this, as demonstrated in Figure S1, we attempted to answer the following 3 questions: What is the relationship between occupational status and depression among middle-aged and older adults in China? How does serving as a state functionary affect this relationship? And how does this relationship differ between urban and rural groups, and between groups with different levels of education?

## 2. Methods

### 2.1. Participants

**Figure 1:**
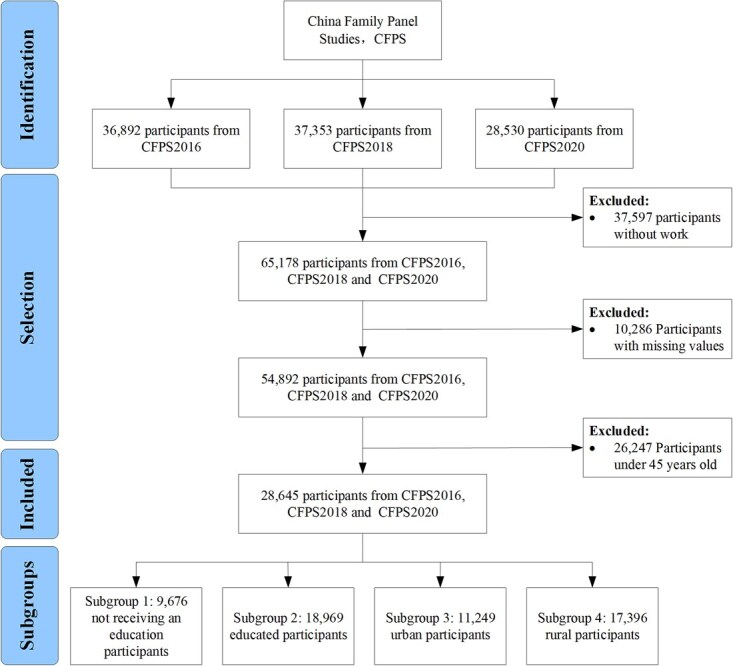
Flow chart of the study participants. Data from the China Family Panel Studies (CFPS) from 2016, 2018, and 2020 were applied.

The participants in this study were from the CFPS, a large tracking survey database covering 25 provinces in China, which collects data for academic research and public policy analysis. The CFPS has new participants joining or participants exiting each year. We used CFPS data for 2016, 2018, and 2020 with participant sizes of 36 892, 37 353, and 28 530, respectively, to form the longitudinal and unbalanced panel data for this study. First, we excluded 37 597 participants who were unemployed using the question “What’s your current working state?” Next, we excluded 10 286 participants with excessive missing values. After excluding 26 247 participants under the age of 45 based on their responses to “What’s your current age?” we finally included 28 645 participants. In addition, we also divided participants into 4 subgroups according to their household registration and education. Subgroups 1, 2, 3, and 4 denote the not receiving an education subgroup, educated subgroup, urban subgroup, and rural subgroup, respectively. Subgroups 1 and 2 included only those participants who were not receiving an education and those who were educated, respectively. Subgroups 3 and 4 contained only urban and rural participants, respectively. Figure 1 illustrates the screening process.

### 2.2. Measures

#### 2.2.1. Dependent variable

Depression: the CFPS uses the Center for Epidemiologic Studies Depression Scale (CES-D) to assess depression. The CES-D scale includes 20 questions about feelings or behaviors.^24^ The higher the participants’ overall scores, the more severe their depressive symptoms.^24^ Therefore, a continuous variable (depression score) was used as the main dependent variable.

#### 2.2.2. Independent variable

Occupational status: we used 3 indicators representing occupational status in the CFPS in this study, namely, the International Socioeconomic Index of Occupational Status (ISEI), the Standard International Occupational Prestige Scale (SIOPS), and the Erikson and Goldthorpe class categories (EGP). CFPS converted the Chinese Standard Classification of Occupations (CSCO) to the International Standard Classification of Occupations (ISCO-88) and obtained these 3 occupation status indicators. CFPS directly provides users with ISEI, SIOPS, and EGP for each participant, while introducing the specific conversion process. The supplementary explanation S1 in the appendix introduces the CFPS official explanation of the construction process of the 3 occupational status indicators. The ISEI was developed from the Socioeconomic Status Index (SEI), which can reflect estimates of the SEI for different occupations; higher ISEI values denote a higher socioeconomic status of the occupation. The SIOPS can reflect people’s overall recognition and subjective evaluation of each occupation, representing occupational prestige, with higher SIOPS values indicating a higher socioeconomic status of the occupation. The EGP uses employment relations to distinguish between different positions of different occupations in the labor market and production units, which can reflect the class of an occupation, with higher EGP values indicating a lower socioeconomic status of the occupation. We used the ISEI, SIOPS, and EGP to comprehensively reflect occupational status from 3 perspectives: socioeconomic status, prestige, and class, respectively.

#### 2.2.3. Focal moderator

State functionary: we measured whether a participant was a state functionary by whether they belonged to *bianzhi*, according to the question proposed in the CFPS, “Do you belong to *bianzhi?*” In China, *bianzhi* is a government organ, public institution, or other public affairs department that confirms the number of staff appointed and is a criterion for judging whether a person is a state functionary.^25^ Thus, state functionary was a binary variable in this study; participants belonging to *bianzhi* were defined as state functionaries and assigned a value of 1 or 0 otherwise.

#### 2.2.4. Covariates

The covariates used in this study included demographic and socioeconomic factors, health status, and living habits. The demographic and socioeconomic factors included sex (male = 1, female = 0), age (age at investigation, continuous), household registration (urban = 1 or rural = 0), home location (set the variables east, west, and middle, respectively; if the participant lived in eastern, middle, or western China, the value of each was 1; otherwise it was 0), family size (number of family members, continuous), marital status (married = 1, unmarried = 0), educational attainment (set the variables separately: not receiving an education, 1-12 years, >12 years; if the education level of the participant fell within the above range, the value was 1; otherwise it was 0), and insurance (“with medical insurance” = 1, “without medical insurance” = 0). Health status factors included self-rated health (very good = 1, fine = 2, not too bad = 3, ordinary = 4, poor = 5), unwell (“being unwell in the past two weeks” = 1, “being well in the past two weeks” = 0), and chronic disease (“with chronic diseases in the past six months” = 1, “without chronic diseases in the past six months” = 0). Living habit factors included exercise (frequency of exercise per week, continuous), smoking (“smoking in the past month” = 1, “no smoking in the past month” = 0), and drinking (“drinking more than three times a week” = 1, “drinking less than three times a week” = 0).

### 2.3. Statistical analysis

This study started with a statistical description of all participants’ characteristics in 2016, 2018, and 2020, focusing on how they changed between 2016 and 2020. Because the CFPS is a tracking survey database and participants enroll or drop out every year, we used a year fixed-effects model with covariates, which is suitable for analyzing unbalanced panel data, to assess the main effect of occupational status on depression. Indicators of occupational status included the ISEI, SIOPS, and EGP. We then included the 2-way interaction term “state functionary × occupational status (ISEI/SIOPS/EGP)” in the year fixed-effects model to evaluate whether being a state functionary had a moderating effect on the association between occupational status and depression. The moderating effects model is superior to heterogeneity analysis in that it can test whether the heterogeneity caused by the moderating variables is significant. According to previous studies, the effects of being a state functionary on the relationship between occupational status and depression and the effect of being a state functionary on depression may be affected by household registration and educational attainment.^8^^,^^18^ China has a strict dual household registration system, in which every Chinese citizen has a clear urban or rural household registration, and a 9-year compulsory education policy. However, before the 9-year compulsory education was universal, a considerable number of citizens still did not receive any education. Therefore, we divided all participants into 4 subgroups—not receiving an education, educated, urban, and rural—again using a year fixed-effects model. We selectively excluded participants in different subgroups. Specifically, in the not receiving an education subgroup, we excluded the educated participants, whereas in the educated subgroup, we retained only the educated participants. Similarly, we also only included participants with urban household registration and participants with rural household registration in the urban and rural subgroups, respectively. When testing regression results in each subgroup, we excluded grouping variables to avoid multicollinearity. For instance, when examining the relationship between occupational status and depression among participants not receiving an education or educated participants, we excluded educational attainment as a covariate. In addition, we tested the regression results using a pooled regression.

## 3. Results

### 3.1. Participants’ characteristics

Table 1 presents a statistical description of the study participants (*n* = 28 645). The average depression score for all participants overall was 33.21, and 32.76, 33.43, and 33.57 in 2016 (*n* = 10 926), 2018 (*n* = 10 288), and 2020 (*n* = 7431), respectively. Participants’ depression scores showed a small increase between 2016 and 2020. In terms of occupational status indicators, participants’ ISEI, SIOPS, and EGP scores were 29.01, 38.46, and 9.01, respectively. From 2016 to 2020, the proportion of state functionaries increased slightly from 4.30% to 4.52% and to 4.62% in 2016, 2018, and 2020, respectively. In addition, from 2016 to 2020, the ISEI and SIOPS showed a small increase, whereas the EGP showed a slight decrease, indicating that participants’ occupational status improved slightly in terms of socioeconomic status, occupational prestige, and class classification. Table 1 also reports participants’ demographic, socioeconomic, and health status factors. Notably, the proportion of not receiving an education individuals decreased from 33.80% in 2016 to 28.40% in 2020. Additionally, the percentage of participants with 1-12 years of education increased from 59.40% in 2018 to 66.90% in 2020. However, the share of participants with more than 9 years of education did not change significantly. Regarding exercise, participants exercised approximately 7 times per week in 2020, an increase from nearly 4 times per week in 2018. Tables S1-S4 also show the statistical description of subgroups.

**Table 1:** Descriptive statistics of all participants from 2014 to 2018.

**Characteristics**	**All participants (*n* = 28 645)**		**Year = 2016 (*n* = 10 926)**		**Year = 2018 (*n* = 10 288)**		**Year = 2020 (*n* = 7431)**	
	**Mean or %**	**SD or *n***	**Mean or %**	**SD or *n***	**Mean or %**	**SD or *n***	**Mean or %**	**SD or *n***
**Depression**	33.21	8.36	32.76	8.33	33.43	8.25	33.57	8.53
**State functionary**	4.46%	1278	4.30%	470	4.52%	465	4.62%	343
ISEI	29.01	11.85	28.73	11.57	29.04	11.83	29.37	12.27
SIOPS	38.46	8.91	38.36	8.93	38.64	8.61	38.36	9.27
EGP	9.01	3.13	9.08	3.12	8.99	3.08	8.92	3.20
**Sex**								
Male	55.00%	15 764	55.20%	6035	54.40%	5597	55.60%	4132
Female	45.00%	12 881	44.80%	4891	45.60%	4691	44.40%	3299
**Age, y**	56.32	8.31	56.02	8.31	56.54	8.39	56.45	8.16
**Urban**	39.30%	11 249	38.50%	4205	39.10%	4022	40.67%	3022
East	39.00%	11 173	39.40%	4308	38.80%	3995	38.62%	2870
West	32.00%	9167	31.50%	3440	32.20%	3315	32.46%	2412
Middle	29.00%	8305	29.10%	3178	29.00%	2978	28.92%	2149
**Family size**	4.11	1.99	4.20	2.00	4.05	1.98	4.09	1.96
**Married**	91.60%	26 231	91.60%	10 011	91.40%	9407	91.68%	6813
**Educational attainment**								
Not receiving an education	33.78%	9676	37.22%	4067	34.03%	3501	28.37%	2108
1-12 y	62.42%	17 880	59.43%	6493	62.39%	6419	66.86%	4968
Above 12 y	3.80%	1089	3.35%	366	3.58%	368	4.77%	355
**With medical insurance**	94.16%	26 972	94.40%	10 314	94.48%	9720	93.37%	6938
**Self-rated health**	3.17	1.24	3.23	1.23	3.18	1.25	3.08	1.25
**Unwell**	33.40%	9578	32.60%	3562	35.59%	3661	31.68%	2355
**With chronic disease**	20.10%	5762	20.20%	2212	20.58%	2217	19.28%	1433
**Exercise**	3.58	3.60	2.03	3.08	2.68	3.43	7.10	1.68
**With smoking habits**	33.80%	9693	34.01%	3716	34.60%	3560	32.53%	2417
**With drinking habits**	19.40%	5566	20.18%	2205	19.90%	2046	17.70%	1315

Abbreviations: EGP, Erikson and Goldthorpe class categories; ISEI, International Socioeconomic Index of Occupational Status; SIOPS, Standard International Occupational Prestige Scale.

### 3.2. Effects of ISEI, SIOPS, and EGP on depression in middle-aged and older adults

Table 2 shows the effect of occupational status on depression among middle-aged and older adults using the ISEI, SIOPS, and EGP. After applying the year fixed-effects model and controlling for covariates, we found that higher ISEI and SIOPS were significantly associated with lower depression scores and higher EGP was significantly associated with higher depression scores. Specifically, for every 1 point increase in ISEI scores, depression scores decreased by 0.03 (coefficient = −0.03; 95% CI, −0.04 to −0.02); for every 1 point increase in SIOPS scores, depression scores decreased by 0.01 (coefficient = −0.01; 95% CI, −0.02 to −0.002); and for every 1 point increase in EGP, depression scores increased by 0.12 (coefficient = 0.12; 95% CI, 0.08 to 0.15). The regression results showed that the higher (lower) the socioeconomic status and occupational prestige of the occupation, the less (more) severe the depression in middle-aged and older individuals. In addition, demographic, socioeconomic, and health status factors significantly impacted individuals’ depression. Table S5 shows the effect of occupational status on depression using pooled regression.

**Table 2:** Association between occupational status and depression among middle-aged and older adults in China.^a^

**Characteristics (*n* = 28 645)**	**ISEI**			**SIOPS**			**EGP**		
	**Coef.**	**SE**	**95% CI**	**Coef.**	**SE**	**95% CI**	**Coef.**	**SE**	**95% CI**
**Occupational status**									
ISEI	−0.03^***^	0.004	(−0.04 to −0.02)						
SIOPS				−0.01*	0.01	(−0.02 to −0.002)			
EGP							0.12^***^	0.02	(0.08 to 0.15)
**Sex**	−1.42^***^	0.12	(−1.65 to −1.19)	−1.46^***^	0.12	(−1.69 to −1.23)	−1.42^***^	0.12	(−1.65 to −1.18)
**Male**	−0.05^***^	0.01	(−0.06 to −0.04)	−0.04^***^	0.01	(−0.05 to −0.03)	−0.05^**^	0.01	(−0.06 to −0.04)
**Urban**	−0.78^***^	0.10	(−0.97 to −0.59)	−0.95^***^	0.10	(−1.14 to −0.77)	−0.73^***^	0.10	(−0.93 to −0.54)
**Region**									
East	−0.50^***^	0.11	(−0.72 to −0.29)	−0.52^***^	0.11	(−0.73 to −0.30)	−0.49^***^	0.11	(−0.71 to −0.28)
West	1.04^***^	0.12	(0.81 to 1.27)	1.08^***^	0.12	(0.86 to 1.31)	1.03^***^	0.12	(0.80 to 1.26)
**Family size**	−0.11^***^	0.02	(−0.15 to −0.06)	−0.10^***^	0.02	(−0.15 to −0.06)	−0.11^***^	0.02	(−0.15 to −0.06)
**Married**	−3.31^***^	0.16	(−3.63 to −2.99)	−3.31^***^	0.16	(−3.63 to −2.99)	−3.32^***^	0.16	(−3.64 to −3.00)
**Educational attainment**									
Not receiving an education	1.23^***^	0.28	(0.68 to 1.78)	1.83^***^	0.27	(1.31 to 2.36)	1.41^***^	0.27	(0.88 to 1.94)
1-12 y	0.14	0.26	(−0.37 to 0.66)	0.66^**^	0.25	(0.17 to 1.16)	0.33	0.25	(−0.16 to 0.82)
**With medical insurance**	−1.35^***^	0.19	(−1.72 to −0.97)	−1.33^***^	0.19	(−1.71 to −0.96)	−1.37^***^	0.19	(−1.74 to −0.99)
**Self-rated health**	1.44^***^	0.04	(1.36 to 1.52)	1.44^***^	0.04	(1.36 to 1.52)	1.44^***^	0.04	(1.36 to 1.52)
**Unwell**	3.15^***^	0.11	(2.94 to 3.35)	3.17^***^	0.11	(2.96 to 3.38)	3.15^***^	0.11	(2.94 to 3.35)
**With chronic disease**	1.11^***^	0.12	(0.87 to 1.34)	1.10^***^	0.12	(0.87 to 1.34)	1.10^***^	0.12	(0.87 to 1.33)
**Exercise**	−0.07^***^	0.02	(−0.10 to −0.04)	−0.07^***^	0.02	(−0.10 to −0.04)	−0.07^***^	0.02	(−0.10 to −0.04)
**With smoking habits**	0.50^***^	0.12	(0.27 to 0.72)	0.50^***^	0.12	(0.27 to 0.73)	0.50^***^	0.12	(0.27 to 0.72)
**With drinking habits**	−0.39^**^	0.12	(−0.63 to −0.15)	−0.39^**^	0.12	(−0.63 to −0.15)	−0.39^**^	0.12	(−0.62 to −0.15)
**Within *R*^2^** ^**b**^	0.1952			0.1940			0.1952		

Abbreviations: Coef., coefficient; EGP, Erikson and Goldthorpe class categories; ISEI, International Socioeconomic Index of Occupational Status; SIOPS, Standard International Occupational Prestige Scale.

a The year fixed-effect model was used and covariates were controlled. ISEI, SIOPS, and EGP were used to represent occupational status. ^*^P < .05, ^**^P < .01, ^***^P < .001.

b This study took fixed effects into account in the model, so we report within R-square.

### 3.3. Moderating effect of being a state functionary on the relationship between occupational status and depression among middle-aged and older adults

**Figure 2:**
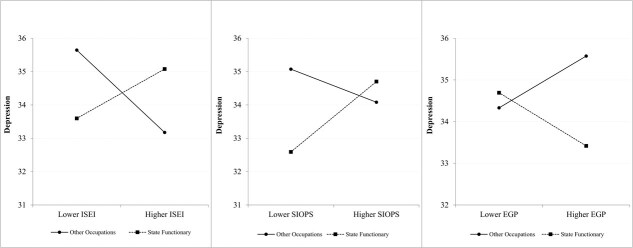
The moderating effect of serving as a state functionary on the relation between occupational status and depression among middle-aged and older individuals.

Using the year fixed-effects model and controlling covariates, we added the 2-way interaction terms “state functionary × occupational status (ISEI/SIOPS/EGP)” and state functionary to the original model to test the moderating effect of being a state functionary on the association between occupational status and depression. As Table 3 demonstrates, the effect of occupational status (ISEI/SIOPS/EGP) on depression remained significant (ISEI: coefficient = −0.03; 95% CI, −0.04 to −0.02; SIOPS: coefficient = −0.02; 95% CI, −0.03 to −0.004; EGP: coefficient = 0.12; 95% CI, 0.09 to 0.16; Table 3) and the 2-way interaction terms “state functionary × occupational status (ISEI/SIOPS/EGP)” were also significant among all middle-aged and older participants (ISEI: coefficient = 0.06; 95% CI, 0.03 to 0.08; SIOPS: coefficient = 0.05; 95% CI, 0.02 to 0.08; EGP: coefficient = −0.25; 95% CI, −0.40 to −0.11; Table 3). As Figure 2 shows, being a state functionary had a significant moderating effect on the relationship between occupational status and depression in middle-aged and older people and even reversed this relationship. More specifically, an increase in ISEI and SIOPS and a decrease in EGP, resulted in more severe depression for middle-aged and older state functionaries.

**Table 3:** Moderating effect of serving as a state functionary on the relation between occupational status and depression among middle-aged and older participants and subgroups.^a^

**Subgroups**	**Characteristics**	**ISEI**			**SIOPS**			**EGP**		
		**Coef.**	**SE**	**95% CI**	**Coef.**	**SE**	**95% CI**	**Coef.**	**SE**	**95% CI**
**All participants (*n* = 28 645)**	ISEI	−0.03^***^	0.004	(−0.04 to −0.03)						
	SIOPS				−0.02^**^	0.01	(−0.03 to −0.004)			
	EGP							0.12^***^	0.02	(0.09 to 0.16)
	State functionary	−3.10^***^	0.73	(−4.53 to −1.67)	−2.90^***^	0.78	(−4.43 to −1.36)	0.61	0.41	(−0.18 to 1.41)
	ISEI × state functionary	0.06^***^	0.01	(0.03 to 0.08)						
	SIOPS × state functionary				0.05^**^	0.02	(0.02 to 0.08)			
	EGP × state functionary							−0.25^**^	0.07	(−0.39 to −0.11)
	**Within *R*^2^** ^**b**^	0.1957			0.1945			0.1956		
**Subgroup 1: Not receiving an education (*n* = 9676)**	ISEI	−0.04^***^	0.02	(−0.07 to −0.01)						
	SIOPS				−0.003	0.01	(−0.03 to 0.03)			
	EGP							0.09	0.05	(−0.002 to 0.18)
	State functionary	−2.07	5.93	(−13.69 to 9.55)	−2.32	5.84	(−13.77 to 9.13)	−1.99	4.02	(−9.86 to 5.89)
	ISEI × state functionary	−0.002	0.13	(−0.26 to 0.25)						
	SIOPS × state functionary				−0.01	0.13	(−0.26 to 0.24)			
	EGP × state functionary							−0.07	0.65	(−1.34 to 1.20)
	**Within *R*^2^** ^**b**^	0.1957			0.1945			0.1956		
**Subgroup 2: Educated (*n* = 18 969)**	ISEI	−0.04^***^	0.004	(−0.05 to −0.03)						
	SIOPS				−0.02^***^	0.01	(−0.03 to −0.01)			
	EGP							0.14^***^	0.02	(0.11 to 0.18)
	State functionary	−3.21^***^	0.70	(−4.59 to −1.84)	−3.03^***^	0.76	(−4.51 to −1.55)	0.49	0.39	(−0.20 to 1.19)
	ISEI × state functionary	0.06^***^	0.01	(0.03 to 0.08)						
	SIOPS × state functionary				0.05^**^	0.02	(0.02 to 0.08)			
	EGP × state functionary							−0.25^***^	0.07	(−0.38 to −0.12)
	**Within *R*^2^** ^**b**^	0.1786			0.1768			0.1787		
**Subgroup 3: Urban (*n* = 11 249)**	ISEI	−0.04^***^	0.01	(−0.05 to −0.03)						
	SIOPS				−0.02*	0.01	(−0.03 to −0.003)			
	EGP							0.14^***^	0.02	(0.10 to 0.19)
	State functionary	−3.34^**^	0.76	(−4.84 to −1.84)	−3.07^***^	0.83	(−4.70 to −1.44)	0.82	0.44	(−0.04 to 1.67)
	ISEI × state functionary	0.06^***^	0.01	(0.03 to 0.09)						
	SIOPS × state functionary				0.06^**^	0.02	(0.02 to 0.09)			
	EGP × state functionary							−0.28^***^	0.08	(−0.43 to −0.13)
	**Within *R*^2^** ^**b**^	0.1772			0.1746			0.1771		
**Subgroup 4: Rural (*n* = 17 396)**	ISEI	−0.03^***^	0.01	(−0.05 to −0.02)						
	SIOPS				−0.01	0.01	(−0.03 to 0.004)			
	EGP							0.11^***^	0.03	(0.05 to 0.16)
	State functionary	−3.18	1.92	(−6.95 to 0.58)	−2.94	1.94	(−6.75 to 0.87)	0.003	0.93	(−1.82 to 1.82)
	ISEI × state functionary	0.05	0.03	(−0.02 to 0.12)						
	SIOPS × state functionary				0.04	0.04	(−0.04 to 0.11)			
	EGP × state functionary							−0.21	0.19	(−0.59 to 0.16)
	**Within *R*^2^** ^**b**^	0.1943			0.1937			0.1943		

Abbreviations: Coef., coefficient; EGP, Erikson and Goldthorpe class categories; ISEI, International Socioeconomic Index of Occupational Status; SIOPS, Standard International Occupational Prestige Scale.

a The year fixed-effect model was used and covariates were controlled. ISEI, SIOPS, and EGP were used to represent occupational status. When testing regression results in each subgroup, grouping variables were excluded as covariates. *P < .05, ^**^P < .01, ^***^P < .001.

b In this study we take fixed effects into account in the model, so we report within R-square.

Table 3 also shows the moderating effect of serving as a state functionary on the relationship between occupational status and depression among middle-aged and older adults in the different subgroups. The relationship between occupational status (ISEI/SIOPS/EGP) and depression was significant in the educated (ISEI: coefficient = −0.04; 95% CI, −0.05 to −0.03; SIOPS: coefficient = −0.02; 95% CI, −0.03 to −0.01; EGP: coefficient = 0.14; 95% CI, 0.11 to 0.18; Table 3) and the urban (ISEI: coefficient = −0.04; 95% CI, −0.05 to −0.03; SIOPS: coefficient = −0.02; 95% CI, −0.03 to −0.003; EGP: coefficient = 0.14; 95% CI, 0.10 to 0.19; Table 3) groups. The 2-way interaction terms “state functionary × occupational status (ISEI/SIOPS/EGP)” were also significant in the educated (ISEI: coefficient = 0.06; 95% CI, 0.03 to 0.08; SIOPS: coefficient = 0.05; 95% CI, 0.02 to 0.08; EGP: coefficient = −0.25; 95% CI, −0.38 to −0.12; Table 2) and urban (ISEI: coefficient = 0.06; 95% CI, 0.03 to 0.09; SIOPS: coefficient = 0.06; 95% CI, 0.02 to 0.09; EGP: coefficient = −0.28, 95% CI, −0.43 to −0.13; Table 2) groups, which means being a state functionary also reverses the relation between occupational status and depression among the middle-aged and older adults. In the not receiving an education and rural groups, the moderating effect of serving as a state functionary on the relationship between occupational status and depression is not significant. Figures S2 and S3 illustrate the moderating effect of being a state functionary on the relationship between occupational status and depression in both the educated and urban subgroups. Table S6 shows the moderating effect of serving as a state functionary using pooled regression.

## 4. Discussion

Using the ISEI, SIOPS, and EGP to indicate occupational status, our results show that the effect of occupational status on depression was significant among middle-aged and older people and that serving as a state functionary is an important moderating variable in this relationship. To the best of our knowledge, this study is the first to assess whether serving as a state functionary has a moderating effect on the relationship between occupational status and depression among middle-aged and older adults. Adopting the year fixed-effects model, we found that middle-aged and older adults with a lower occupational status were more depressed. After adding the 2-way interaction term “state functionary × occupational status (ISEI/SIOPS/EGP),” we discovered that serving as a state functionary reversed the relations between occupational status and depression. Specifically, middle-aged and older state functionaries with higher occupational status were more depressed. Serving as a state functionary had the same effect in the educated and urban groups but not in the not receiving an education and rural groups.

Our study confirms the view that high occupational status is associated with low levels of depression in China among middle-aged and older adults.^8^^,^^9^ The underlying reason could be that people with lower occupational status may perceive themselves as lacking promotional prospects and remain in a more insecure work environment for a long time, leading to poorer mental health.^9^^,^^10^ In addition, low occupational status may predict downward social mobility,^8^ which may contribute to a sense of injustice.^26^ Social class divisions may also produce differences in mental health,^27^ leading to more severe depression in people of lower social classes.^28^ What is more, jobs with a lower occupational status may involve more physical labor and higher physical demands on practitioners.^29^ Additionally, compared with those holding jobs with a higher occupational status, practitioners with lower occupational status have less disease screening and prevention,^30^ which may result in them neglecting their physical and mental diseases. Meanwhile, people with higher occupational status have more resources (eg, higher incomes and greater autonomy at work) that help them buffer against all kinds of possible stress.^31^ These studies confirm our findings and contribute to understanding why low occupational status is correlated with more severe depressive symptoms among middle-aged and older adults in China.

Our study also demonstrates that being a state functionary may result in a positive relationship between higher occupational status and greater depressive symptoms because state functionaries with higher ranks may have to endure multiple stressors that offset the rewards of higher occupational status.^14^ First, owing to the nature of the work of state functionaries, as they rise in rank they often face heavier workloads and greater social responsibility and develop more exclusive relationships with their work.^32^ This is accompanied by a blurring of the line between work and non-work, and work stress seeping into daily life.^14^ At the same time, with the modernization of China, the working mode of the government has also shifted from a bureaucracy to a democracy and from simplicity to pluralism,^33^ which has led to substantial changes in the working environment, including increased work pressure and stricter supervision by public opinion.^19^ Owing to China’s special administrative system, high-level state functionaries often face more complex working environments, including more complicated interpersonal relationships and fierce competition for positions,^34^ all of which may contribute to their greater susceptibility to depression. Furthermore, although it has undergone long-term reform, the performance and compensation management system of state functionaries still requires further improvement.^35^ Owing to the flawed compensation system, many state functionaries complain that the incentive mechanism is not working and that putting in more effort does not result in receiving substantial rewards such as higher pay.^20^ In addition, to adapt to the complex work environment, state workers have to perform tasks against their will, which can lead to health-risk habits such as excessive smoking and drinking, insomnia, and no exercise.^20^^,^^36^ Impaired physical health may also worsen depressive symptoms. Our findings extend the current conclusions of extant literature on the relationship between occupational status and depression.

In addition, our study shows that the moderating effect of being a state worker is significant only in the educated and urban subgroups and not in the not receiving an education or rural subgroups, which may hint at negative outcomes for those who are not receiving an education and those who reside in urban areas. First, for participants in the educated subgroup, higher education may lead to higher average pay and autonomy compared with participants in the not receiving an education group.^14^ However, for state functionaries in the educated subgroup, the specific nature of their work may offset the rewards of high occupational status and mean that they benefit less from high occupational status than other participants in the educated subgroup. For participants in the not receiving an education subgroup, overall pay and autonomy are not as high; therefore, the negative effects of being a state functionary are less significant. In addition, state functionaries in educated groups may face greater competition and higher peer pressure, potentially exacerbating the negative effects of being state functionaries.^13^

At the same time, owing to a higher degree of marketization, living in cities entails a higher cost of living than living in the countryside; however, participants living in cities generally have higher income levels.^37^ However, the income gap between different regions and different levels of national workers is smaller, which means that compared with other participants, urban state functionaries face greater living pressure due to the imbalance between income levels and living costs.^18^ Excessive economic pressure may also lead to depression.^38^ State functionaries in the urban subgroup are more likely to face issues (eg, increased competition, high uncertainty, and blurred boundaries between work and non-work), which, in turn, may lead to more significant negative effects.^9^ The greater number and complexity of public affairs in cities is also likely to lead to greater work stress than that experienced by rural state workers, leading to greater depression.^18^

This study has several limitations. First, the CES-D is a self-rating scale and thus may lead to bias.^39^ Second, the participants included in this study may have had multiple occupations. Although occupational status (ISEI, SIOPS, and EGP) in the CFPS is measured according to the participant’s primary occupation, we are concerned that the diversity of occupations may have introduced errors into the study results.^9^ In the future, we plan to explore the influence of different occupational statuses in multiple occupations on depression. Third, some variables that have been proven to regulate the effect of occupational status on depression were not examined in this study, such as the frequency of internet use,^8^ because they are not included in the CFPS. Future studies should consider these variables to improve the conclusions drawn in this study. Finally, some studies have examined the heterogeneity of samples based on provinces but this study does not.^18^ Because the different provinces in these studies represent different degrees of marketization, this study examines the heterogeneity of urban and rural participants; however, to some extent, it can also reflect the impact of different degrees of marketization on the heterogeneity of the research results.

### 4.1. Conclusion

Using CFPS data for 2016, 2018, and 2020, this study adopted a year fixed-effects model and found a significant association between high occupational status and less severe depression among middle-aged and older adults. The study also revealed that being a state functionary may result in a positive relationship between higher occupational status and greater depressive symptoms among middle-aged and older adults. The moderating effect of state functionality was significant only in the educated and urban subgroups. To actively respond to population aging, more attention needs to be paid to the mental health of middle-aged and older adults with lower occupational status. For state functionaries with higher occupational status, attention needs to be paid to their mental health and improving their mental health awareness.

## Supplementary Material

Web_Material_uiaf008

## Data Availability

The original data presented in the study are publicly available. These data can be found here: http://www.isss.pku.edu.cn/cfps/.
