# Supplementary material for: What makes reversal: examining the moderating effect of being a state functionary on occupational status and depression among middle-aged and older people in China
Source: J Occup Health. 2025 Mar 25;67(1):uiaf008. doi: 10.1093/joccuh/uiaf008 (PMC11952907; doi:10.1093/joccuh/uiaf008)
Supplement: Web_Material_uiaf008 [file web_material_uiaf008.zip › Appendix.doc]

**Supplemental Materials**

**What Makes Reversal: Examining the Moderating Effect of Being a State Functionary on Occupational Status and Depression Among Middle-Aged and Older People in China**

**Catalogue**

**Supplementary explanation:** The construction method of three types of occupational status indicators and the specific conversion process.	1

**Table S1** The descriptive statistics of all participants from 2014 to 2018 in Illiteracy group.	3

**Table S2** The descriptive statistics of all participants from 2014 to 2018 in educated group.	4

**Table S3** The descriptive statistics of all participants from 2014 to 2018 in urban group.	5

**Table S4** The descriptive statistics of all participants from 2014 to 2018 in rural group.	6

**Table S5** The association between occupational status and depression among middle aged and the older in China.	7

**Table S6** The moderating effect of serving as a state functionary on the relation between occupational status and depression among the middle-aged and the older in all participants and subgroups.	8

**Figure S1** Conceptual framework diagram: The relation between occupational status and depression was examined, and whether serving as a state functionary had a moderating effect on the relation. The same tests were also carried out among subgroups with different educational attainment and household registration.	9

**Figure S2** The moderating effect of serving as a state functionary on the relation between occupational status and depression among the middle-aged and the older in educated group.	10

**Figure S3** The moderating effect of serving as a state functionary on the relation between occupational status and depression among the middle-aged and the older in urban group.	11

**Supplementary explanation:** The construction method of three types of occupational status indicators and the specific conversion process.

1) At present, the most commonly used codes in academic research are ISCO88 and ISCO68 provided by the International Labor Organization (ILO), both of which adopt a four-level coding system, the first level is the main category, the second level is the secondary category, the third level is the unit category, and the fourth level is the occupation name. Chinese census also develops local occupational Classification and code systems, Chinese Standard Classification of Occupations (CSCO), including CSCO99 and CSCO09. CSCO09 and CSCO99 are three-level codes, the first level is a large class, the second level is a middle class, and the third level is a small class. The CFPS occupational classification is based on the CSCO09 code system with changes and adjustments. CFPS converts (CSCO) to International Standard Classification of Occupation (ISCO-88), Based on ISCO-88 Occupational classification code, The International Socio-Economic Index of Occupational Status (ISEI) and The Standard International are constructed The Occupational Prestige Scale (SIOPS), and the Occupational classification codes for adult questionnaire respondents (Erikson and Goldthorpe's Class Categories, EGP).
2) CFPS introduces the detailed process of conversion in the official website, including code matching, missing value processing, and data conversion. For the specific conversion process, please refer to the CFPS official website:

https://www.isss.pku.edu.cn/cfps/cjwt/zy/1356889.htm

https://www.isss.pku.edu.cn/cfps/cjwt/zy/1356890.htm

http://isss.pku.edu.cn/cfps/docs/20180927133140517170.pdf

3) Reference books on occupational status classification

Duncan, Otis Dudley. 1961. “A Socioeconomic Index for All Occupations.” Pp.109-138 in Occupations and Social Status, edited by Albert J. Reiss, Jr. New York: Free Press.

Erikson, Robert and John H. Goldthorpe. 1992. The Constant Flux: A Study of Class Mobility in Industrial Societies. Oxford University Press, USA.

Erikson, Robert, John H. Goldthorpe, and Lucienne Portocarero. 1979. “Intergenerational Class Mobility in Three Western European Societies: England, France, and Sweden.” British Journal of Sociology 30: 415-441.

Ganzeboom, Harry B., Paul M. De Graaf, and Donald J. Treiman. 1992. “A Standard International Socio-Economic Index of Occupational Status.” Social Science Research 21:1-56.

Ganzeboom, Harry B., Paul M. De Graaf, and Donald J. Treiman. 1996. “Internationally Comparable Measures of Occupational Status for the 1988 International Standard Classification of Occupations.” Social Science Research 25: 201-239.

Treiman, Donald J. 1977. Occupational Prestige in Comparative Perspective. New York: Academic Press.

ILO(International Labour Organization). 1990. International Standard Classification of Occupations: ISCO-88. Geneva, Switzerland: International Labour Organization.

**Table S1 The descriptive statistics of all participants from 2014 to 2018 in Illiteracy group.**

| **Characteristics** | **All Participants** | **Year = 2016** | **Year = 2018** | **Year = 2020** |
| --- | --- | --- | --- | --- |
|  | **N = 9676** | **N = 4067** | **N= 3501** | **N= 2108** |
| **Depression** | 35.00 | 34.54 | 35.27 | 35.44 |
| **State functionary** | 0.20% | 0.20% | 0.10% | 0.20% |
| **Occupational status** |  |  |  |  |
| ISEI | 24.69 | 24.74 | 24.78 | 24.44 |
| SIOPS | 38.24 | 38.16 | 38.43 | 38.09 |
| EGP | 10.22 | 10.21 | 10.19 | 10.31 |
| **Sex** |  |  |  |  |
| Male | 37.60% | 39.20% | 36.30% | 36.40% |
| Female | 62.40% | 60.80% | 63.70% | 63.60% |
| **Age** | 59.30 | 58.91 | 59.47 | 59.79 |
| **Urban** | 27.00% | 26.40% | 27.30% | 27.50% |
| **Region** |  |  |  |  |
| East | 30.60% | 31.50% | 30.60% | 28.90% |
| West | 43.40% | 42.40% | 43.70% | 45.00% |
| Middle | 26.00% | 26.10% | 25.70% | 26.10% |
| **Family Size** | 4.35 | 4.42 | 4.27 | 4.34 |
| **Married** | 87.90% | 87.80% | 87.90% | 88.10% |
| **With insurance** | 93.40% | 94.00% | 93.80% | 91.50% |
| **Self-rated health** | 3.33 | 3.39 | 3.31 | 3.24 |
| **Unwell** | 41.30% | 40.30% | 43.70% | 39.20% |
| **With chronic disease** | 23.60% | 23.40% | 23.90% | 23.30% |
| **Exercise** | 3.07 | 1.49 | 2.24 | 7.47 |
| **With smoking habits** | 26.20% | 27.10% | 26.10% | 24.80% |
| **With drinking habits** | 14.90% | 15.80% | 15.00% | 13.10% |

**Table S2** The descriptive statistics of all participants from 2014 to 2018 in educated group.

| **Characteristics** | **All Participants** | **Year = 2016** | **Year = 2018** | **Year = 2020** |
| --- | --- | --- | --- | --- |
|  | **N = 18969** | **N = 6859** | **N= 6787** | **N= 5323** |
| **Depression** | 32.30 | 31.70 | 32.48 | 32.84 |
| **State functionary** | 6.60% | 6.70% | 6.80% | 6.40% |
| **Occupational status** |  |  |  |  |
| ISEI | 31.21 | 31.09 | 31.24 | 31.32 |
| SIOPS | 38.57 | 38.48 | 38.75 | 38.46 |
| EGP | 8.39 | 8.41 | 8.37 | 8.36 |
| **Sex** |  |  |  |  |
| Male | 63.90% | 64.70% | 63.70% | 63.20% |
| Female | 36.10% | 35.30% | 36.30% | 36.80% |
| **Age** | 54.80 | 54.31 | 55.02 | 55.13 |
| **Urban** | 45.60% | 45.70% | 45.20% | 45.90% |
| **Region** |  |  |  |  |
| East | 43.30% | 44.10% | 43.10% | 42.50% |
| West | 26.20% | 25.00% | 26.30% | 27.50% |
| Middle | 30.50% | 30.90% | 30.60% | 30.00% |
| **Family Size** | 3.99 | 4.06 | 3.93 | 3.99 |
| **Married** | 93.40% | 93.90% | 93.30% | 93.10% |
| **With insurance** | 94.60% | 94.70% | 94.80% | 94.10% |
| **Self-rated health** | 3.10 | 3.13 | 3.12 | 3.02 |
| **Unwell** | 29.40% | 28.00% | 31.40% | 28.70% |
| **With chronic disease** | 18.40% | 18.40% | 18.90% | 17.70% |
| **Exercise** | 3.84 | 2.35 | 2.91 | 6.95 |
| **With smoking habits** | 37.70% | 38.10% | 39.00% | 35.60% |
| **With drinking habits** | 21.70% | 22.80% | 22.40% | 19.50% |

**Table S3** The descriptive statistics of all participants from 2014 to 2018 in urban group.

| **Characteristics** | **All Participants** | **Year = 2016** | **Year = 2018** | **Year = 2020** |
| --- | --- | --- | --- | --- |
|  | **N = 11249** | **N = 4205** | **N= 4022** | **N= 3022** |
| **Depression** | 32.15 | 31.76 | 32.33 | 32.46 |
| **State functionary** | 9.54% | 9.23% | 9.82% | 9.60% |
| **Occupational status** |  |  |  |  |
| ISEI | 33.86 | 33.54 | 33.92 | 34.23 |
| SIOPS | 38.05 | 37.81 | 38.28 | 38.10 |
| EGP | 7.52 | 7.57 | 7.57 | 7.40 |
| **Sex** |  |  |  |  |
| Male | 56.40% | 56.60% | 56.10% | 56.40% |
| Female | 43.60% | 43.40% | 43.90% | 43.60% |
| **Age** | 54.98 | 54.65 | 55.26 | 55.05 |
| **Region** |  |  |  |  |
| East | 45.60% | 46.30% | 45.60% | 44.50% |
| West | 24.30% | 23.50% | 24.10% | 25.80% |
| Middle | 30.10% | 30.20% | 30.30% | 29.70% |
| **Family Size** | 3.94 | 3.96 | 3.88 | 3.98 |
| **Married** | 91.90% | 91.90% | 91.90% | 92.00% |
| **Educational attainment** |  |  |  |  |
| not receiving an education | 23.20% | 25.50% | 23.80% | 19.20% |
| 1-12 years | 68.20% | 66.80% | 68.10% | 70.40% |
| Above 12years | 8.60% | 7.70% | 8.10% | 10.40% |
| **With medical insurance** | 93.70% | 93.60% | 93.90% | 93.50% |
| **Self-rated health** | 3.09 | 3.13 | 3.11 | 3.02 |
| **Unwell** | 30.00% | 28.90% | 31.90% | 29.00% |
| **With chronic disease** | 18.10% | 18.60% | 18.20% | 17.40% |
| **Exercise** | 3.78 | 2.55 | 2.87 | 6.70 |
| **With smoking habits** | 32.80% | 33.30% | 33.90% | 30.80% |
| **With drinking habits** | 21.10% | 21.70% | 22.10% | 18.80% |

**Table S4** The descriptive statistics of all participants from 2014 to 2018 in rural group.

| **Characteristics** | **All Participants** | **Year = 2016** | **Year = 2018** | **Year = 2020** |
| --- | --- | --- | --- | --- |
|  | **N = 17396** | **N = 6721** | **N= 6266** | **N= 4409** |
| **Depression** | 33.90 | 33.38 | 34.14 | 34.34 |
| **State functionary** | 1.20% | 1.20% | 1.10% | 1.20% |
| **Occupational status** |  |  |  |  |
| ISEI | 25.87 | 25.71 | 25.92 | 26.03 |
| SIOPS | 38.72 | 38.70 | 38.87 | 38.54 |
| EGP | 9.96 | 10.03 | 9.90 | 9.96 |
| **Sex** |  |  |  |  |
| Male | 54.20% | 54.40% | 53.30% | 55.10% |
| Female | 45.80% | 45.60% | 46.70% | 44.90% |
| **Age** | 57.19 | 56.88 | 57.36 | 57.41 |
| **Region** |  |  |  |  |
| East | 34.80% | 35.10% | 34.50% | 34.60% |
| West | 37.00% | 36.50% | 37.50% | 37.00% |
| Middle | 28.20% | 28.40% | 28.00% | 28.40% |
| **Family Size** | 4.23 | 4.34 | 4.16 | 4.16 |
| **Married** | 91.30% | 91.40% | 91.10% | 91.40% |
| **Educational attainment** |  |  |  |  |
| not receiving an education | 40.60% | 44.50% | 40.60% | 34.70% |
| 1-12 years | 58.70% | 54.80% | 58.70% | 64.40% |
| Above 12years | 0.70% | 0.70% | 0.70% | 0.90% |
| **With medical insurance** | 94.50% | 94.90% | 94.80% | 93.30% |
| **Self-rated health** | 3.23 | 3.29 | 3.23 | 3.12 |
| **Unwell** | 35.70% | 34.90% | 38.00% | 33.50% |
| **With chronic disease** | 21.40% | 21.30% | 22.10% | 20.60% |
| **Exercise** | 3.45 | 1.70 | 2.57 | 7.37 |
| **With smoking habits** | 34.50% | 34.40% | 35.10% | 33.70% |
| **With drinking habits** | 18.40% | 19.20% | 18.50% | 16.90% |

**Table S5 The association between occupational status and depression among middle aged and the older in China.**

| **Characteristics** | **ISEI** | | **SIOPS** | | **EGP** | |
| --- | --- | --- | --- | --- | --- | --- |
|  | **Coef.** | **95%CI** | **Coef.** | **95%CI** | **Coef.** | **95%CI** |
| **Occupational Status** |  |  |  |  |  |  |
| ISEI | -0.03*** | (-0.04, -0.02) |  |  |  |  |
| SIOPS |  |  | -0.01* | (-0.02, -0.003) |  |  |
| EGP |  |  |  |  | 0.12*** | (0.09, 0.15) |
| **Sex** | -1.43*** | (-1.66, -1.20) | -1.47*** | (-1.70, -1.24) | -1.43*** | (-1.66, -1.20) |
| **Male** | -0.05*** | (-0.06, -0.04) | -0.04*** | (-0.05, -0.03) | -0.05*** | (-0.06, -0.04) |
| **Urban** | -0.79*** | (-0.98, -0.60) | -0.97*** | (-1.15, -0.78) | -0.74*** | (-0.94, -0.55) |
| **Region** |  |  |  |  |  |  |
| East | -0.50*** | (-0.71, -0.28) | -0.51*** | (-0.73, -0.30) | -0.49*** | (-0.70, -0.27) |
| West | 1.07*** | (0.84, 1.30) | 1.11*** | (0.88, 1.34) | 1.06*** | (0.83, 1.29) |
| **Family Size** | -0.11*** | (-0.16, -0.07) | -0.10*** | (-0.15, -0.06) | -0.11*** | (-0.16, -0.07) |
| **Married** | -3.32*** | (-3.64, -3.00) | -3.32*** | (-3.64, -3.00) | -3.33*** | (-3.65, -3.01) |
| **Educational attainment** |  |  |  |  |  |  |
| not receiving an education | 1.18*** | (0.63, 1.73) | 1.78*** | (1.25, 2.30) | 1.36*** | (0.83, 1.89) |
| 1-12 years | 0.12 | (-0.39, 0.63) | 0.64* | (0.15, 1.14) | 0.31 | (-0.18, 0.81) |
| **With medical insurance** | -1.37*** | (-1.74, -0.99) | -1.35*** | (-1.73, -0.98) | -1.39*** | (-1.76, -1.01) |
| **Self-rated health** | 1.43*** | (1.35, 1.51) | 1.44*** | (1.36, 1.52) | 1.43*** | (1.35, 1.51) |
| **Unwell** | 3.16*** | (2.95, 3.37) | 3.18*** | (2.97, 3.39) | 3.16*** | (2.95, 3.37) |
| **With chronic disease** | 1.07*** | (0.84, 1.31) | 1.07*** | (0.84, 1.31) | 1.07*** | (0.83, 1.30) |
| **Exercise** | 0.02 | (-0.001, 0.05) | 0.02 | (-0.003, 0.05) | 0.02 | (-0.001, 0.05) |
| **With smoking habits** | 0.50*** | (0.27, 0.72) | 0.50*** | (0.27, 0.73) | 0.50*** | (0.27, 0.73) |
| **With drinking habits** | -0.41** | (-0.65, -0.18) | -0.41** | (-0.65, -0.17) | -0.41** | (-0.65, -0.17) |

Notes:

1) *P<0.05, **P<0.01, ***P<0.001. Coef., coefficient.
2) The pooled regression was used and covariates were controlled.
3) ISEI, SIOPS and EGP was used to represent occupational status.

**Table S6** The moderating effect of serving as a state functionary on the relation between occupational status and depression among the middle-aged and the older in all participants and subgroups.

| **Subgroups** | **Characteristics** | **ISEI** | | **SIOPS** | | **EGP** | |
| --- | --- | --- | --- | --- | --- | --- | --- |
|  |  | **Coef.** | **95%CI** | **Coef.** | **95%CI** | **Coef.** | **95%CI** |
| **All Participants**  **(N = 28645)** | ISEI | -0.04*** | (-0.04, -0.03) |  |  |  |  |
|  | SIOPS |  |  | -0.02** | (-0.03, -0.01) |  |  |
|  | EGP |  |  |  |  | 0.13*** | (0.09, 0.16) |
|  | State Functionary | -3.22*** | (-4.66, -1.79) | -3.02*** | (-4.56, -1.48) | 0.55 | (-0.25, 1.34) |
|  | ISEI×State Functionary | 0.06*** | (0.03, 0.08) |  |  |  |  |
|  | SIOPS×State Functionary |  |  | 0.05** | (0.02, 0.08) |  |  |
|  | EGP×State Functionary |  |  |  |  | -0.26*** | (-0.40, -0.11) |
| **Subgroup 1:**  **not receiving an education (N=9676)** | ISEI | -0.04* | (-0.07, -0.01) |  |  |  |  |
|  | SIOPS |  |  | -0.004 | (-0.03, 0.02) |  |  |
|  | EGP |  |  |  |  | 0.09* | (0.003, 0.18) |
|  | State Functionary | -1.99 | (-13.63, 9.65) | -2.40 | (-13.87, 9.07) | -2.23 | (-10.12, 5.66) |
|  | ISEI×State Functionary | -0.005 | (-0.26, 0.25) |  |  |  |  |
|  | SIOPS×State Functionary |  |  | -0.01 | (-0.26, 0.24) |  |  |
|  | EGP×State Functionary |  |  |  |  | -0.04 | (-1.31, 1.23) |
| **Subgroup 2:**  **Educated**  **(N=18969)** | ISEI | -0.04*** | (-0.05, -0.03) |  |  |  |  |
|  | SIOPS |  |  | -0.02** | (-0.03, -0.01) |  |  |
|  | EGP |  |  |  |  | 0.14*** | (0.10, 0.18) |
|  | State Functionary | -3.37*** | (-4.75, -1.99) | -3.22*** | (-4.70, -1.73) | 0.64 | (-0.12, 1.41) |
|  | ISEI×State Functionary | 0.06*** | (0.03, 0.09) |  |  |  |  |
|  | SIOPS×State Functionary |  |  | 0.53** | (0.02, 0.08) |  |  |
|  | EGP×State Functionary |  |  |  |  | -0.27*** | (-0.41, -0.14) |
| **Subgroup 3:**  **Urban**  **(N=11249)** | ISEI | -0.04*** | (-0.05, -0.03) |  |  |  |  |
|  | SIOPS |  |  | -0.02* | (-0.03, -0.003) |  |  |
|  | EGP |  |  |  |  | 0.15*** | (0.10, 0.19) |
|  | State Functionary | -3.47*** | (-4.98, -1.97) | -3.20*** | (-4.83, -1.57) | 0.78 | (-0.07, 1.64) |
|  | ISEI×State Functionary | 0.06*** | (0.03, 0.09) |  |  |  |  |
|  | SIOPS×State Functionary |  |  | 0.06** | (0.02, 0.09) |  |  |
|  | EGP×State Functionary |  |  |  |  | -0.29*** | (-0.44, -0.14) |
| **Subgroup 4:**  **Rural**  **(N=17396)** | ISEI | -0.03*** | (-0.05, -0.02) |  |  |  |  |
|  | SIOPS |  |  | -0.02 | (-0.03, 0.003) |  |  |
|  | EGP |  |  |  |  | 0.11*** | (0.05, 0.16) |
|  | State Functionary | -3.07 | (-6.83, 0.70) | -2.89 | (-6.71, 0.93) | -0.16 | (-1.99, 1.66) |
|  | ISEI×State Functionary | 0.04 | (-0.02, 0.11) |  |  |  |  |
|  | SIOPS×State Functionary |  |  | 0.03 | (-0.04, 0.11) |  |  |
|  | EGP×State Functionary |  |  |  |  | -0.20 | (-0.57, 0.18) |

Notes:

1)	*P<0.05, **P<0.01, ***P<0.001. Coef., coefficient.

2)	The pooled regression was used and covariates were controlled.

3)	ISEI, SIOPS and EGP was used to represent occupational status.

**Figure S1** Conceptual framework diagram: The relation between occupational status and depression was examined, and whether serving as a state functionary had a moderating effect on the relation. The same tests were also carried out among subgroups with different educational attainment and household registration.

**Figure S2** The moderating effect of serving as a state functionary on the relation between occupational status and depression among the middle-aged and the older in educated group.

**Figure S3** The moderating effect of serving as a state functionary on the relation between occupational status and depression among the middle-aged and the older in urban group.
